# Human-biting behaviour of *Leptoconops irritans* (Diptera: Ceratopogonidae) in a touristic area of the Balearic Islands (Spain)

**DOI:** 10.1007/s00436-024-08447-z

**Published:** 2025-01-30

**Authors:** Mikel Alexander González, Carlos Barceló, Aroa Rodríguez-López, Jordi Figuerola, Miguel Ángel Miranda

**Affiliations:** 1 Doñana Biological Station–Spanish Research Council (EBD-CSIC), Seville, Spain; 2 CIBER Epidemiology and Public Health (CIBER ESP), Madrid, Spain; 3 Applied Zoology and Animal Conservation Group, University of the Balearic Islands (ZAP-UIB), Palma, Spain; 4 Institute for Agro-Environmental Research and Water Economics (INAGEA), University of the Balearic Islands, Palma, Spain

**Keywords:** Biting midges, Coastal dune, Diurnal pest, Landing preferences, Mediterranean islands, Menorca

## Abstract

Biting midges of genus *Leptoconops* Skuse 1889 are small blood-feeding insects recognized as highly irritating diurnal pests in certain regions around the globe. In Europe, their presence is poorly documented, except in France and Italy. Following reports of human discomfort in a tourist area of Menorca, Balearic Islands (Spain), a small-scale study was conducted to identify the biting species and assess their preferred biting sites using a human-landing assay along a habitat gradient in a coastal dune area. *Leptoconops irritans* (Noé, 1905) was identified based on morphological features and DNA barcoding. This species reached high densities (average rates of 3.3 landings/min), particularly near coastal dune vegetation. No statistically significant differences were found among the four main body sites for landings of *L. irritans* (*F*_3,6.023_ = 1.80, *p* = 0.250): head (*n* = 91, 53.8%), lower extremities (*n* = 39, 23.1%), upper extremities (*n* = 37, 21.9%), and other covered areas (*n* = 2, 1.2%). Landing preferences varied among the three volunteers, and bites progressed differently. This study represents the second documented case of *Leptoconops* midges causing human discomfort in Spain. We hope this research will stimulate further interest in this understudied genus, which has been largely overlooked across much of Europe.

## Introduction

*Leptoconops* Skuse 1889 is a genus of tiny, blood-sucking midges belonging to the family Ceratopogonidae. These are notorious for their painful bites, which can cause significant irritation and allergic reactions in humans and animals (Kettle 1962). *Leptoconops* midges are considered as troublesome as other members of the same family (e.g., *Culicoides*) in terms of direct nuisance to humans, such as *Culicoides impunctatus* Goetghebuer 1920 in Europe or *Culicoides furens* (Poey 1853) in the Americas (Carter 192; Linley and Davies 1971; Blackwell et al. 1994). However, *Culicoides* biting midges are better known due to their role as vectors of several pathogens of medical and veterinary importance (Kettle 1977). Knowledge about *Leptoconops* in Europe is limited. Most research on this genus has focused on its biology and ecology in Italy and France, mainly published between the 1960s and the 1980s (Fausto et al. 2007).

In Spain, only a few local studies have confirmed the presence of *Leptoconops* in specific regions such as Catalonia, Andalusia, Aragón, and the Basque Country (Ortiz and Ordeix 2009; Delecolle 1999; González et al. 2013; Obregon et al. 2019), but its distribution and potential public health impact remain largely understudied. For this reason, it is reasonable to hypothesize that this genus is more widely distributed. This lack of awareness may lead to their presence going unnoticed or their nuisance being misidentified with other groups, such as Simuliidae (black flies), which are also small, dark-coloured flies that produce painful bites. Enhanced awareness and targeted research are essential to fully understand the distribution and impact of *Leptoconops* in Spain.

This study was initiated following a personal communication from Dr. Albert Bertolero, who reported small insects aggressively biting him while he was conducting turtle sampling in the dunes of Cala Tirant (Menorca, Spain). Our aim was to confirm the identity of the human-biting species and gather additional information on its biting preferences.

## Material and methods

**Fig. 1:**
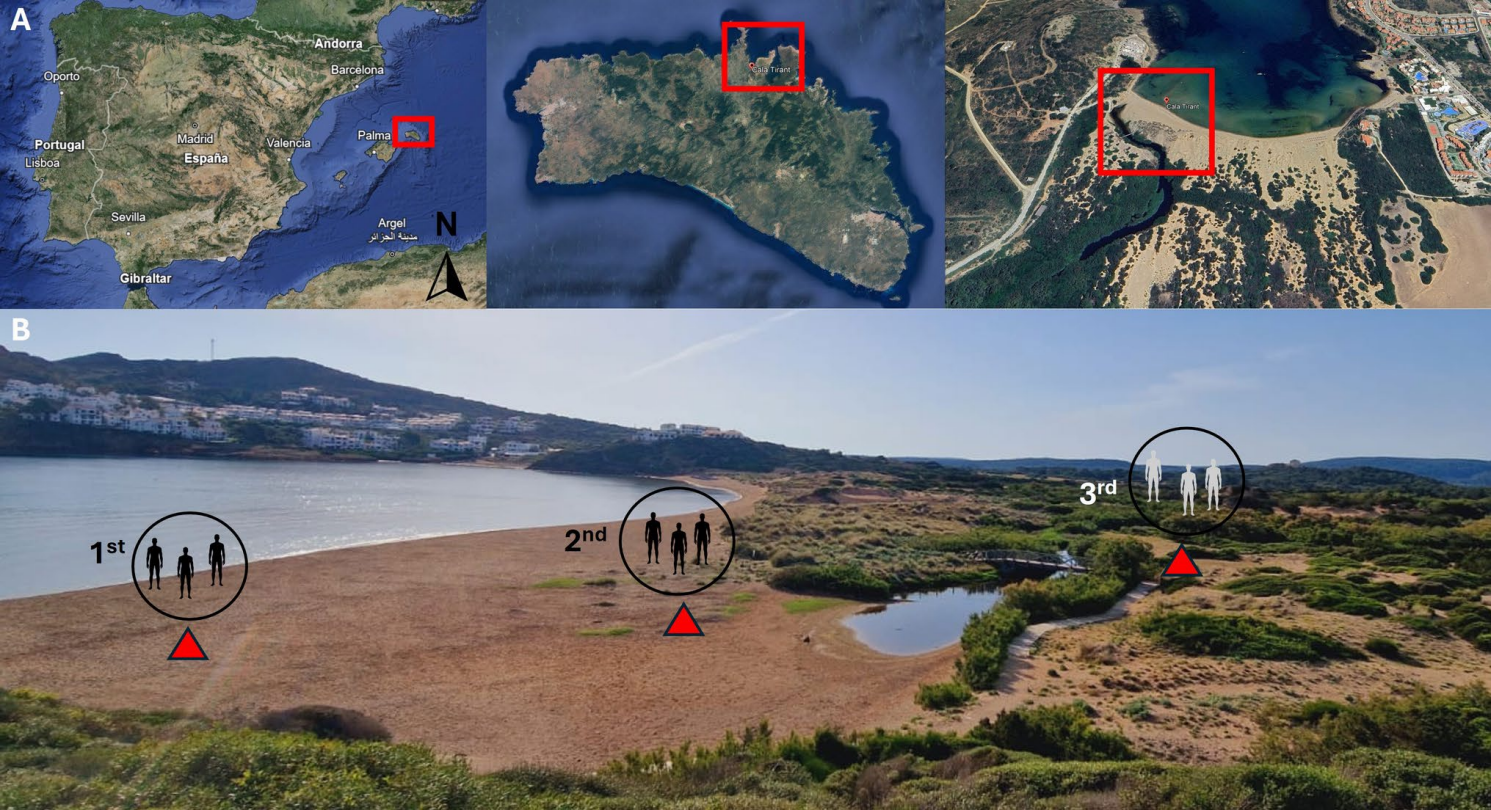
Location and setup of the human-landing assay at Cala Tirant, Menorca, Spain. **A** Map showing the location of Cala Tirant on the north coast of Menorca (Balearic Islands, Spain) within the Mediterranean Sea, highlighted by the red box. **B** The three sampling points selected for the human-landing assay: the first point (1st position) was located 5 m from the seashore, the second point (2nd position) near the beginning of the dune area, and the third point (3rd position) behind the marsh

A small-scale study was conducted on June 7, 2023, at Cala Tirant (40° 02′ 39.7″ N, 4° 06′ 10.1″ E), in the municipality of Es Mercadal, on the north coast of the island of Menorca (Balearic Islands, Spain) (Fig. 1A). Cala Tirant is a 600 m-wide cove located in a large bay, characterized by fine golden sand and a dune system with a continuous gradient of vegetation from the first dunes to the second dunes (foredunes). A nearby marsh drains into the area, forming a marsh at the back of the cove. The surrounding landscape features low hills with bushes and tamarisk trees. There are also a few apartments, bars, and houses situated on both sides of the cove. The climate of the area, according to the Emberger classification, is arid-semi humid. During the summer months, the average temperature typically ranges from 25 to 30 °C, and precipitation is quite low, usually ranging from 5 to 15 mm per month (Pons et al. 2017).

Three locations were selected for a human-landing assay, set along a gradient from the seashore to the back of the cove (0–300 m) (Fig. 1B). The first location (position 1) was 5 m from the seashore, the second (position 2) was at the edge of the dune area, and the third (position 3) was behind the torrent in the *Tamarix* sp. vegetation zone, with approximately 100 m between each point (Fig. 1B). Three researchers volunteered as human bait to assess the landings of *Leptoconops* midges. In random order, one of the volunteers stood upright with his legs (from knee to feet) and arms (from shoulders) exposed for 3 min, while the second volunteer collected insects landing using a mouth-operated entomological aspirator, and the remaining volunteer recorded the human-landing activity. Afterwards, the other two volunteers took turns being exposed at the same location. Once the first round in the three positions was completed, the same round was repeated once, alternating the order of the volunteers. This process was repeated at the second and third sampling points. Specimens that remained perched for at least 3 s were aspirated and counted as valid. For each detected landing, the number of insects and general position on the body where the biting midges perched was noted for the uncovered areas of the body (head-neck, upper extremities, and lower extremities). Landings on covered areas, such as the back, were discarded.

The participants were all men with ages comprised between 38 and 53 years. On the day of the trial, none of the participants used cologne or any product that might modify human skin odour. The first round was conducted at 7:15 a.m. (Tª 17–18 °C), and the second at 8:15 a.m. (Tª 18–20 °C) on a sunny day with light winds. Additionally, the vegetation of the area was sampled for 10 min using a sweep net (at ground level and striking the vegetation) to determine whether the midges were resting in those areas.

Specimens were stored in 70% ethanol and transported to the laboratory for species identification. A subsample of *Leptoconops* (*n* = 5) was mounted on slides and identified using previously published keys (Carter 1921). A 658 bp fragment of the cytochrome oxidase subunit I (COI) gene was amplified and sequenced in three specimens following the procedures described in González et al. (2024a, b) with a modification in the amplification reaction. It started with a polymerase activation phase at 94 °C for 4 min followed by 35 cycles at 94 °C for 1 min, 50 °C for 1 min (previously set at 40 °C), and 72 °C for 1 min, followed by a final extension for 10 min at 72 °C. Voucher specimens were stored in the Department of Zoology of the University of the Balearic Islands.

Landing rates were calculated as the number of landings divided by the time of exposure for the three operators (pooled). A generalized linear model (GLM) with normal distributed errors was used to analyse differences in the landings of *Leptoconops* spp. The number of captures was log10-transformed. Volunteer and the interaction between volunteer identity and the site of the landing were included as random factors, while the study area and site of the landing were included as fixed factors. The statistically significant interaction between volunteer identity and the site of landing was interpreted with slice tests. All analyses were done in JMP 9.0 (SAS Institute).

## Results and discussion

**Fig. 2:**
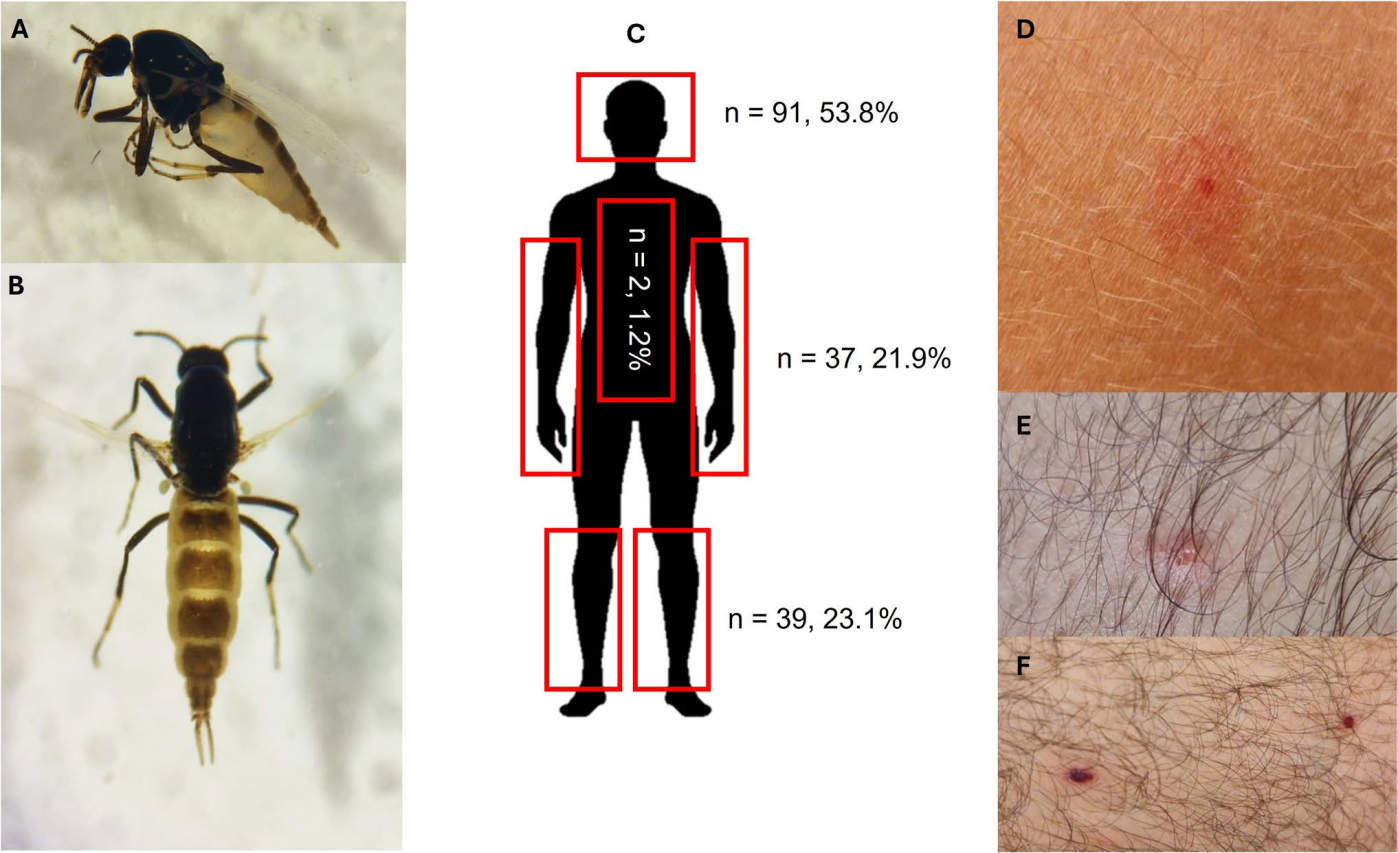
Morphology, landing preferences, and bite reactions of *Leptoconops irritans* midges. **A**, **B** Close-up of *L. irritans* in dorsal view (bottom) and lateral view (top). **C** Diagram showing the preferred landing sites on the human body, with highlighted regions indicating areas of highest landing frequency. The number of landings is expressed for each body part. **D–F** Bites on the body of one of the volunteers. Appearance a few minutes after being bitten (**D**), several days after (**E**), and small skin lesions that persisted and remained itchy for 15 days (**F**)

All the specimens collected on human volunteers were identified as *Leptoconops irritans* (Noé, 1905) females (*n* = 169). No specimens were collected by sweeping the vegetation. *Leptoconops irritans* can be easily separated from the remaining *Leptoconops* species by the characteristic isabella-coloured abdomen, the elongated proboscis (largely exceeding the head length), and the unique long and slender palpus, among others (Fig. 2A, B). In Spain, this species has only been recorded in the region of Catalonia (Ortiz and Ordeix 2009); thus, this record represents the second report in the country. High-quality sequences of COI were obtained from two out of the three sequenced specimens (GenBank accession numbers: LC858744 and LC858745) and showed 98.04–98.53% of homology with the *L. irritans* sequences from Italy (OM672386-90–91–92–93). Both sequences differed in three nucleotides.

No statistical differences were found between body sites of *L. irritans* landing (*F*_3,6.023_=1.80; *p*=0.250) but showed significant differences within the volunteers (*F*_3,63.74_; *p*<0.001). Overall, most of the landings occurred on the head-neck, followed by lower extremities and upper extremities and few landings in other body sites (Fig. 2C). Specifically, the three sites of the body with more landings were the head (*n*=74), the calves (*n*=21), and the back neck (*n*=10). However, such preferences depended on the volunteer. For example, the head was the preferred site selected by two volunteers (0.77 ± 0.10 and 0.68 ± 0.09), but the legs were the preferred site by the third volunteer (0.50 ± 0.10). In the absence of current studies on trophic preferences, previous research has suggested that *Leptoconops* species have an affinity to feed on the head and arms of humans, causing nuisance by crawling in the hair, beneath clothes, and in the ears (Carter 1921), which aligns with our field observations. In fact, we observed *Leptoconops* having a high tendency to fly around the head; they hovered around and quickly passed through the hair to reach the scalp, remaining well fixed on the skin (the mouth aspirator was unable to remove them once they were fixed). *Leptoconops irritans* bites are noticeable and painful, resembling those of black flies, with a small puncture wound (red dot) at the centre of the papule, which is typical of telmophagic feeding insects (Fig. 2D). Bites from *Leptoconops* can cause varied skin reactions, ranging from mild, itchy, pink-red lesions that resolve quickly to severe symptoms with intense itching, notable oedema, and polymorphic conditions that can progress to impetiginized or bullous lesions with serous or sero-hemorrhagic content (ADOI 2024). Among the three volunteers, only one developed small skin lesions that persisted and remained itchy for 15 days (Harant and Galan 1944) (Fig. 2E, F).

This *Leptoconops* species is well known in Italy, where it is thought to be widely distributed, particularly in the coastal plains. Locally called “serafiche”, these tiny midges are extremely bothersome when they occur in large numbers during their peak activity period, gaining a notorious evil reputation and limiting the use of certain areas as tourist attractions and recreational locations (Carter 1921; Bettini 1968; Polidori et al. 2023). Despite the notable flight activity of *L. irritans* observed at the coast dunes during the study, it is unlikely to be a significant problem as their populations diminish throughout the summer (Carter 1921). Their peak activity occurs early in the morning, around 8:00 a.m. (Carrieri et al. 2011), rather than at midday when tourists are more likely to sunbathe.

Significant differences were found along the habitat gradient (*F*_2,58.2_ = 8.55; *p* = 0.0006). A lower number of *L. irritans* were collected at the seashore (position 1) compared to dune habitats (*p* < 0.001), and no differences were found between position 2 and position 3 (*t*_58.16_ = 0.29; *p* = 0.770). A higher number of landings were recorded in position 3 (4.7 landings/min), followed by position 2 (4.1 landings/min) and position 1 (0.9 landing/min). In the absence of more detailed information and based on previous descriptions of its habitat, it is plausible that its immature stage development is linked to the permanent marsh (torrent) at the back of the beach. In Europe, this species is frequent in coastal environments, along with *Leptoconops kerteszi* Kieffer, 1908. The latter breeds in coastal habitats on sandy shores devoid of vegetation and kept constantly damp due to the capillarity rise of saltwater (Majori et al. 1970; Raspi et al. 2007), while *L. irritans* can be also found both near the sea or in soils further inland, specifically in calcareous, halomorphic soils that crack polygonally upon drying (Rioux et al. 1968; Bettini 1968; Carrieri et al. 2011).

In conclusion, our study has confirmed the presence of *L. irritans* in Spain and provides new COI sequence data that enhances our understanding of this species. By documenting its biting behaviour and site preferences, particularly its strong tendency to target the head and extremities, we provide valuable insights into its interactions with human hosts. This research underscores the importance of continued local investigations to better understand and manage *L. irritans* and its impact on human activities. By expanding knowledge of this understudied genus within Spain, we aim to encourage further research in other regions where its presence has been noted but remains unexplored.

## Data Availability

No datasets were generated or analysed during the current study.

## References

[CR1] ADOI (Associazione Dermatologi-Venereologi Ospedalieri Italiani e della Sanità Pubblica) (2024) Patologia cutanea da puntura insetti. https://www.adoi.it/patologia-cutanea-da-puntura-insetti/. Accessed 17 Jun 2024

[CR2] Bettini EF (1968) *Leptoconops irritans* Noé in Grossetano: problems in control. Rev di Parassitol 29(1):33–47 5744490

[CR3] Blackwell A, Mordue (Luntz) AJ, Mordue W (1994) Identification of bloodmeals of the Scottish biting midge, *Culicoides impunctatus*, by indirect enzyme-linked-immunosorbent-assay (Elisa). Med Vet Ent 8:20–24. 10.1111/j.1365-2915.1994.tb00378.x 10.1111/j.1365-2915.1994.tb00378.x 8161839

[CR4] Carrieri M, Montemurro E, Valentino SV et al (2011) Influence of environmental and meteorological factors on the biting activity of *Leptoconops noei* and *Leptoconops irritans* (Diptera: Ceratopogonidae) in Italy. J Am Mosq Control Assoc 27:30–38. 10.2987/8756-971X-27.1.30 21476445 10.2987/8756-971X-27.1.30

[CR5] Carter HF (1921) A revision of the genus *Leptoconops*, Skuse. Bull Entomol Res 12:1–28. 10.1017/S0007485300044813

[CR6] Delecolle J (1999) Ceratopogonidés (Diptera, Nematocera) do Los Monegros. Bol La Soc Entomol Aragon 24:137

[CR7] Fausto AM, Belardinelli MC, Cocchi M et al (2007) Le serafiche (*Leptoconops* spp., Diptera: Ceratopogonidae) nelle aree umide del litorale grossetano: aspetti biologici e socio-sanitari. Atti Acc Naz It Entomol 55:79–83

[CR8] González MA, López S, Goldarazena A (2013) New record of the biting midge *Leptoconops noei* in Northern Spain: notes on its seasonal abundance and flying height preference. J Insect Sci 13:1–10. 10.1673/031.013.4501 23909239 10.1673/031.013.4501 PMC3740921

[CR9] González MA, Magallanes S, Bravo-Barriga D et al (2024a) Sampling of *Culicoides* with nontraditional methods provides unusual species composition and new records for southern Spain. Parasite Vectors 17:1–14. 10.1186/s13071-024-06414-2 10.1186/s13071-024-06414-2 PMC11318182 39135087

[CR10] González MA, Ruiz-Arrondo I, Magallanes S et al (2024b) Molecular and morphological analysis revealed a new *Lipoptena* species (Diptera: Hippoboscidae) in southern Spain harbouring *Coxiella burnetii* and bacterial endosymbionts. Vet Parasitol 332:110300. 10.1016/j.vetpar.2024.110300 39270602 10.1016/j.vetpar.2024.110300

[CR11] Harant H, Galan G (1944) Notes sur les Diptères de la région méditerranéenne VII. Remarques sur les *Leptoconops*: *Leptoconops lisbonnei* n. sp. Bull Soc Pathol Exot 37:170–172

[CR12] Kettle DS (1962) The bionomics and control of *Culicoides* and *Leptoconops* (Diptera, Ceratopogonidae = Heleidae). Annu Rev Entomol 7(1):401–408

[CR13] Kettle DS (1977) Biology and bionomics of bloodsucking ceratopogonids. Annu Rev Entomol 22(1):33–51 319742 10.1146/annurev.en.22.010177.000341

[CR14] Linley JR, Davies JB (1971) Sandflies and tourism in Florida and the Bahamas and Caribbean Area. J Econ Entomol 64:264–278. 10.1093/jee/64.1.264

[CR15] Majori S, Bettini E, Finizio GP (1970) Research on Ceratopogonidae in the province of Grosseto. IV. Identification of the breeding places of *Leptoconops* (*Holoconops*) *kerteszi* Kieffer, 1908. Riv Parassitol 31:279–84 5523702

[CR16] Obregon R, Flores E, Jordano D (2019) First report of the Asian tiger mosquito, *Aedes* (*Stegomyia*) *albopictus* Skuse, 1984 (Diptera, Culicidae) in Cordoba (southern Spain). new challenges for the administration and citizens of Cordoba. J Eur Mosq Control Assoc 37:29–33

[CR17] Ortiz J, Ordeix M (2009) Espiadimonis, nàiades, sabaters i cuques de capsa. Els macroinvertebrats dels rius i zones humides de Catalunya. Centre d’Estudis dels Rius Mediterranis – Museu del Ter – Eumo editorial, Vic.

[CR18] Polidori C, Gabrieli P, Arnoldi I et al (2023) Morphological and molecular insights into the diversity of *Leptoconops* biting midges from a heavily infested Mediterranean area. Curr Res Parasitol Vector-Borne Dis 4:100142. 10.1016/j.crpvbd.2023.100142 37822789 10.1016/j.crpvbd.2023.100142 PMC10562859

[CR19] Pons GX, Martín-Prieto J, Mir-Gual M et al (2017) Los sistemas dunares costeros de Menorca. Monogr La Soc D’historia Nat Les Balear 25:87–110

[CR20] Raspi A, Canovai R, Loni A, Santini L (2007) *Leptoconops* (*Holoconops*) *kerteszi* Kieffer (Diptera: Ceratopogonidae) in the coastal area of Grosseto: Eeo-ethological aspects. Bull Insectology 60:1–6

[CR21] Rioux JA, Descous S, Corre JJ et al (1968). Ecologie de *Leptoconops irritans* Noé, 1905 en moyenne Camargue. Localisation et dynamique des biotopes larvaires. Rev Écol (La Terre et La Vie) 22(4): 458–469.

